# Comparison of older and newer generations of ActiGraph accelerometers with the normal filter and the low frequency extension

**DOI:** 10.1186/1479-5868-10-51

**Published:** 2013-04-25

**Authors:** Kelli L Cain, Terry L Conway, Marc A Adams, Lisa E Husak, James F Sallis

**Affiliations:** 1 Department of Psychology, San Diego State University, San Diego, CA, USA; 2 Department of Family and Preventive Medicine, University California, San Diego, San Diego, CA, USA; 3 School of Nutrition and Health Promotion, Arizona State University, Phoenix, AZ, USA

**Keywords:** Data processing, Physical activity, Sedentary, Methods, Step counts, Measurement, GT3X

## Abstract

**Background:**

Many studies used the older ActiGraph (7164) for physical activity measurement, but this model has been replaced with newer ones (e.g., GT3X+). The assumption that new generation models are more accurate has been questioned, especially for measuring lower intensity levels. The low-frequency extension (LFE) increases the low-intensity sensitivity of newer models, but its comparability with older models is unknown. This study compared step counts and physical activity collected with the 7164 and GT3X + using the Normal Filter and the LFE (GT3X+N and GT3X+LFE, respectively).

**Findings:**

Twenty-five adults wore 2 accelerometer models simultaneously for 3Âdays and were instructed to engage in typical behaviors. Average daily step counts and minutes per day in nonwear, sedentary, light, moderate, and vigorous activity were calculated. Repeated measures ANOVAs with post-hoc pairwise comparisons were used to compare mean values. Means for the GT3X+N and 7164 were significantly different in 4 of the 6 categories (pâ€‰<â€‰.05). The GT3X+N showed 2041 fewer steps per day and more sedentary, less light, and less moderate than the 7164 (+25.6, -31.2, -2.9 mins/day, respectively). The GT3X+LFE showed non-significant differences in 5 of 6 categories but recorded significantly more steps (+3597 steps/day; pâ€‰<â€‰.001) than the 7164.

**Conclusion:**

Studies using the newer ActiGraphs should employ the LFE for greater sensitivity to lower intensity activity and more comparable activity results with studies using the older models. Newer generation ActiGraphs do not produce comparable step counts to the older generation devices with the Normal filter or the LFE.

## Background

Accelerometers are widely accepted as valid objective measures of physical activity, and the ActiGraph is the most commonly used brand. There have been several models of ActiGraphs distributed since 1993: old generation models such as the 7164 and new generation models such as the GT1M, GT3X and the GT3X+. While both generations record accelerations on the vertical axis, they contain different types of internal mechanisms. The 7164, which was often referred to as the CSA or MTI, contains a uniaxial piezoelectric cantilever beam sensor that detects dynamic accelerations resulting from motion [1,2]. The new generation models were introduced in 2005 and contain a Micro-Electro-Mechanical-System (MEMS) capacitive accelerometer capable of detecting both static and dynamic accelerations in either two or three axes [1]. ActiGraph also introduced a new digital filtering algorithm (referred to as the Normal filter) for use with the new models that was designed to eliminate any acceleration noise outside of the normal human activity frequency bandwidth [2,3].

Although the old models have been discontinued and replaced with new generation devices, many population-based physical activity studies [4-6] used the 7164. A review of 183 youth accelerometer studies from 2005â€“2010 revealed that the 7164 was used in 80% of studies reporting an ActiGraph model [7]. Considering the majority of physical activity studies to date have used an old generation ActiGraph, the comparability of the data collected with the old and new generation ActiGraphs needs to be established. This is relevant for between study comparisons (e.g., comparing prevalence rates to national estimates) and within study comparisons (e.g., longitudinal studies examining changes over time) when different instruments (i.e., generations of devices) were used.

The 7164 model has shown to be a valid device for detecting steps across different walking speeds (<1.5% error) compared with direct observation [8]. A recent validation study questions the assumption that the newer models are more accurate measurement devices than older models. Feito and colleagues found that the 7164 was more accurate in detecting steps compared with the new generation models when using direct observation as the criterion measure in the lab and a validated pedometer in free-living settings [9].

Laboratory studies have shown that old and new generation models are generally comparable for moderate-to-vigorous physical activity, but that the new ActiGraphs require higher acceleration to record non-zero counts, making them less sensitive to low intensity movement [10-12]. With a growing interest in sedentary behavior [13], this is an important concern. In response to these findings, ActiGraph introduced a Low-Frequency Extension (LFE) that can be applied to data collected with the new models to expand the range of the Normal filtering algorithm on the lower end of the intensity threshold (to increase sensitivity to low intensity movement) [14]. Two studies have examined the LFE filter with the GT3X model and found that it results in significantly different activity estimates across all intensity levels and step counts compared to the Normal filter [15] and while the LFE attenuates differences between older and newer generation devices in lower intensity activity, it creates a bias in moderateâ€“intensity physical activity estimates [16]. The purpose of the present study was to compare activity intensity and step count data collected with the older 7164 to data collected with the newer GT3X + using both the Normal filter and the LFE filter in free-living adults.

## Methods

This study was approved by the Institutional Review Board at San Diego State University. This was a convenience sample of twenty-five adults (mean ageâ€‰=â€‰32.8; SDâ€‰=â€‰11.3; 52% female; 20% Hispanic/non-white). The response rate was 83.3%. Participants were eligible if they were able to engage in moderate physical activity and willing to wear the devices. Participants wore an old and new ActiGraph (7164 and GT3X+) on the same belt (separated by 10 millimeters) around the waist for 3 complete days (mixture of weekdays and weekend days). The order of the devices on the belt was randomized, and participants were instructed to wear the monitors centered on their right hip during all waking hours (except when swimming or bathing). Participants were asked to engage in their normal daily routine while wearing the accelerometers.

Calibration was checked on the 7164 models pre- and post-data collection using the CAL71 device distributed by ActiGraph and found to be within the acceptable range [17]. According to ActiGraph [18], the GT3X + does not require calibration. The 7164 monitors were initialized with a 60-second epoch and data from the GT3X + were converted to 60Âseconds post-download. For this study, only step counts and data from the vertical axis were compared.

When accelerometers were returned, data from both models were downloaded and screened for device malfunction [19]. Data from the GT3X + monitors were downloaded two times, once with the Normal filter and once with the LFE applied. Thus, there were 3 data sets: 7164, GT3X + with the Normal filter (GT3X+N), and GT3X + with the LFE (GT3X+LFE). Days with at least 8 valid wearing hours (nonwear defined as â‰¥60Âminutes of consecutive zero counts in the vertical axis) were processed using MeterPlus v4.3 software [20]. Sedentary was defined as â‰¤100 counts per minute (cpm) and Freedson adult cut points [21] were used to define light (101â€“1951Âcpm), moderate (1952â€“5724Âcpm; 3â€“5.9 METS), and vigorous activity (5725+ cpm; â‰¥6 METS). These commonly used cut points were derived from the 7164 model and therefore appropriate when comparability with the existing literature is a key concern. Minute-by-minute data were summarized into daily averages for step counts and activity intensity categories. Repeated measures ANOVAs with post-hoc pairwise comparisons were used to compare mean values for each category across the 3 data sets and Bland-Altman plots were created to assess the limits of agreement.

## Findings

The final sample included a total of 75Âdays of data across 25 participants with an average daily wear time of 12.2Âhours (SDâ€‰=â€‰1.7). There was complete agreement between the 7164, GT3X+N and GT3X+LFE on classifying days as valid so each dataset contained the same 75 wearing days. All participants were included in final analyses as no monitor problems were detected. Results of the comparisons for step counts and activity categories can be found in FigureÂ1. The GT3X+N showed 2041 fewer step counts per day compared with the 7164 (CI _95% diff:_ -2944.0, -1138.7; pâ€‰<â€‰.001; Panel 1). The GT3X+N also showed 25.6Âmin/day more sedentary (CI _95% diff_: 12.9, 38.2; pâ€‰<â€‰.001; Panel 3), 31.2Âmin/day less light (CI _95% diff_: -37.6, -24.7; pâ€‰<â€‰.001; Panel 4) and 2.9Âmin/day less moderate activity (CI _95% diff_: -4.9, -0.8; pâ€‰<â€‰.05; Panel 5) compared with the 7164. The differences in nonwear time (+8.3Âmin/day) and vigorous activity (+0.2Âmin/day) were non-significant (CI _95% diff_: -0.9, 17.4 and âˆ’0.8, 1.6; Panels 2 and 6). The GT3X+LFE showed 29.1Âmin/day less sedentary (CI _95% diff_: -34.4, -23.8; pâ€‰<â€‰.001; Panel 3), 26.8Âmin/day more light (CI _95% diff_: 21.9, 31.7; pâ€‰<â€‰.001; Panel 4), and 2.6Âmin/day more moderate activity (CI _95% diff_: 1.7, 3.4; pâ€‰<â€‰.001; Panel 5) compared with the GT3X+N. The differences between the GT3X+LFE and the 7164 were non-significant in all activity intensity categories (+7.8Âmin/day nonwear (CI _95% diff_: -1.4, 17.0); -3.5Âmin/day sedentary (CI _95% diff_: -15.9, 8.9); -4.3Âmin/day light (CI _95% diff_: -11.5, 2.9); -0.3Âmin/day moderate (CI _95% diff_: -2.8, 2.1); and 0.4Âmin/day vigorous (CI _95% diff_: -0.8, 1.6). However, the GT3X+LFE showed 5638 and 3597 more step counts compared with the GT3X+N and the 7164, respectively (CI _95% diff_: 4532.4, 6743.8 and 2994.4, 4198.9; pâ€‰<â€‰.001; Panel 1). The Bland-Altman plots demonstrated acceptable limits of agreement for nonwear, sedentary, light, moderate and vigorous activity between the GT3X+LFE and 7164 (see FigureÂ2, Panels 1-5). The results showed identical patterns after adjusting for age, gender and device number as covariates.

**Figure 1:**
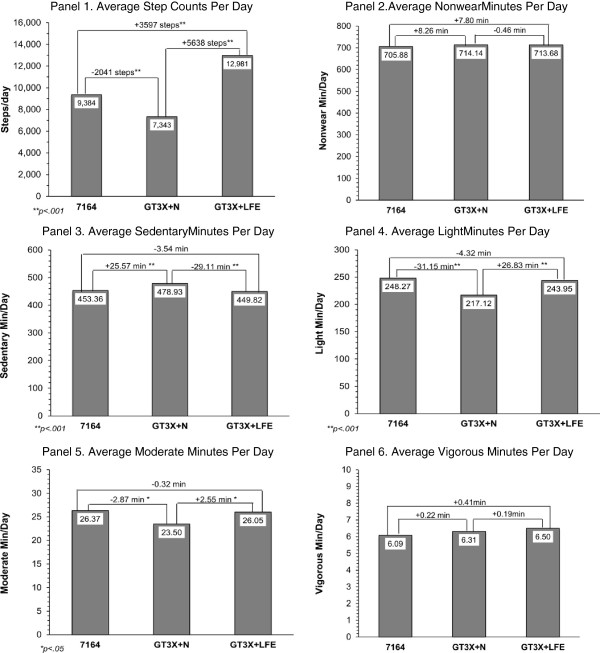
**Activity and step count comparisons between 7164 and GT3X + with the Normal Filter and LFE.** Panels 1-6.

**Figure 2:**
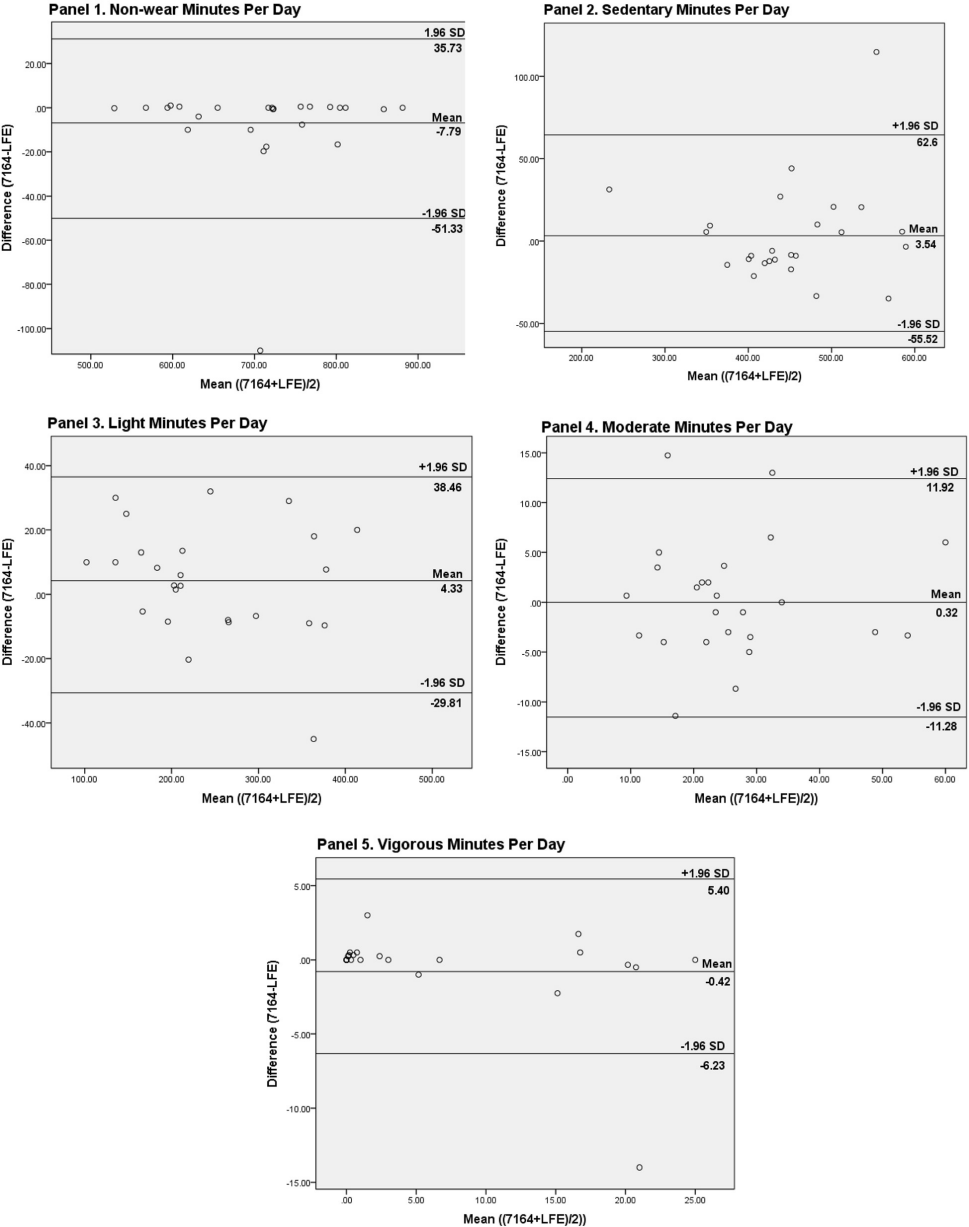
**Bland-Altman plots for nonwear, sedentary, light, moderate and vigorous activity.** Panels 1-5.

## Discussion

Significant differences in step counts, sedentary, light, and moderate activity were detected between the older 7164 and newer GT3X + accelerometers using the Normal filter. These differences limit the comparability of sedentary behavior and physical activity results across or within studies using different generations of ActiGraphs. The differences between the 7164 and GT3X+N ranged from 2.9Âminutes/day with moderate activity, 25.6Âminutes/day with sedentary behavior, and 31.2Âminutes/day with light activity. The application of the LFE with the new generation ActiGraph reduced these differences to non-significant levels (0.3Âmin/day in moderate, 3.5Âmin/day in sedentary and 4.3Âmin/day in light), and the 95% confidence intervals suggested that true differences larger than 2.8, 15.9 or 11.5 mins/day, respectively, were unlikely. However, the LFE did not reduce the difference in step counts, which were about 2000 fewer steps with the GT3X+N and about 3600 more steps with the GT3X+LFE compared with the validated 7164 [8,9]. Bland-Altman plots showing reasonable agreement between the GT3X+LFE and 7164 in nonwear, sedentary, light, moderate and vigorous activity support the conclusion that the data from these devices are fairly interchangeable.

The results showing more sedentary and less light activity with the GT3X+N compared with the 7164 are consistent with laboratory and free-living studies showing the newer generation models to be less sensitive on the lower end of the intensity spectrum compared with the older model [10-12,15,16]. The results in the moderate and vigorous intensity categories are less consistent with previous studies. Present results showing less moderate activity with the GT3X+N compared with the 7164 are not consistent with studies showing no significant differences between newer and older generation devices [10-12,16]. In the present study, the LFE reduced the differences in moderate intensity while Mathias and colleagues [16] found that the LFE introduced moderate-intensity differences. However, Wanner and colleagues [15] found about 3Âminutes per day more moderate-to-vigorous physical activity with the GT3X-LFE compared to the GT3X-N which is similar to the present findings. The present study showed no significant differences in vigorous activity between the 7164 and GT3X + with either the Normal or LFE filter which is not consistent with the findings of Mathias and colleagues showing more vigorous with the 7164 compared to the GT3X with both the Normal and LFE filters [16]. Different study designs (laboratory vs free-living setting), data processing methods, and Actigraph models (GT3X vs GT3X+) may account for the inconsistent results across studies.

The documented differences between models (and using different filters) call into question the generalizability of findings from calibration/validation studies to data collected with different generation ActiGraphs. For example, cut points derived using the 7164 model (e.g., <100Âcpm, Freedson [21]) may not be suitable for data collected with the GT3X+N. The validity concerns that arise from applying 7164-derived cut points to GT3X+N data are attenuated when using the LFE filter. Similarly, algorithms and cut points derived from studies using the newer models (with the Normal filter) [22-24] may not be appropriate for data collected with the 7164 model or a newer model with the LFE. It may be necessary to develop cut points and nonwear definitions that are model and filter-specific.

This study had several strengths. The use of the older generation Actigraph (7164) allowed us to explore the implications of comparing physical activity estimates produced by older and newer generation devices; the majority of studies to date that have used the 7164 model. The 7164 models in this study were selected from a batch of over 100 devices and we ensured proper calibration and no malfunction. Also, using the GT3X + model (instead of the GT3X) allowed for the filter to be applied post-data collection on the same data thereby reducing inter-monitor variability that could have been introduced if two GT3X devices were used to compare the filters. To our knowledge, this study is the first to compare step counts between older and newer generation Actigraphs. Wanner and colleagues compared free-living step counts in 65 adults and found significantly more step counts recorded with the LFE compared to the Normal filter, a finding consistent with the present study. However, the 7164 model was not included in that study. Limitations of the present study included a small sample so meaningful but non-significant differences between models may have occurred, although data were collected at all intensities and significant differences were detected for small differences. The convenience sample may limit the generalizability of results.

Studies using a newer generation ActiGraph should employ the LFE filter for greater sensitivity to lower intensity activity, more comparable results to studies using the older models, and more appropriate application of established calibration cut points. Longitudinal studies that change from an older generation to a newer generation ActiGraph, or that use older and newer generation models simultaneously, should employ the LFE filter for more comparable activity estimates across models. Studies using a newer generation ActiGraph and interested in measuring step counts will either significantly underestimate steps using the GT3X + (Normal filter) or significantly overestimate steps using the GT3X + with the LFE. There is evidence that the older generation ActiGraph (7164) is the most accurate at detecting steps compared with the new ActiGraphs, and some validated pedometers [9]. No solution was identified to create comparable step counts across Actigraph generations. Present findings need to be replicated in other populations, such as children and older adults, who have different movement patterns.

## Abbreviations

LFE: Low Frequency Extension; Cpm: Counts per minute

## Competing interests

The authors declare that they have no competing interests.

## Authors' contributions

KC: conceived of study, participated in the study design and coordination, conducted analyses, drafted the manuscript, and approved the final manuscript as submitted. TC: participated in conceptualizing the study design and data analysis, reviewed and revised the manuscript, and approved the final manuscript as submitted. MA: participated in data analysis, reviewed and edited the manuscript, and approved the final manuscript as submitted. LH: participated in study coordination and data management and approved the final manuscript as submitted. JS: Reviewed and edited the manuscript approved the final manuscript as submitted. All authors read and approved the final manuscript.
